# Protocol for detecting genome-wide introgressed genes and evaluating their functional legacy

**DOI:** 10.1016/j.xpro.2026.104553

**Published:** 2026-05-19

**Authors:** Zhenbin Jiao, Zhiyao Ren, Chao Hu, Xiaokai Ma, Guo-Qiang Zhang, Li-Jun Chen, Gang Wei, Dong-Hui Peng, Yi-Bo Luo, Siren Lan, Zhong-Jian Liu

**Affiliations:** 1 Key Laboratory of Orchid Conservation and Utilization of National Forestry and Grassland Administration at College of Landscape Architecture and Art, Fujian Agriculture and Forestry University, Fuzhou, Fujian 350002, China; 2 State Key Laboratory of Systematic and Evolutionary Botany, Institute of Botany, Chinese Academy of Sciences, Beijing 100093, China; 3 University of Chinese Academy of Sciences, Beijing 100049, China; 4 Institute of Gerontology, Guangzhou Geriatric Hospital, Guangzhou Medical University, Guangzhou, Guangdong 510550, China; 5 Eastern China Conservation Centre for Wild Endangered Plant Resources, Shanghai Chenshan Botanical Garden, Shanghai 201602, China; 6 Center for Genomics and Biotechnology, Haixia Institute of Science and Technology, School of Future Technology, Fujian Agriculture and Forestry University, Fuzhou, Fujian 350002, China; 7 Shenzhen Key Laboratory for Orchid Conservation and Utilization and The National Orchid Conservation Center of China, The Orchid Conservation and Research Center of Shenzhen, Shenzhen, Guangdong 518114, China; 8 School of Pharmaceutical Sciences, Guangzhou University of Chinese Medicine, Guangzhou, Guangdong 510006, China

**Keywords:** Genetics, Plant sciences, Evolutionary biology

## Abstract

Introgressed genes can significantly influence the ecological fate of recipient species exposed to novel environments. Here, we present a protocol for detecting introgressed genes and assessing their functional impact in recipient species by combining *f*_dM_ statistics with expression profiling under various stress conditions. We describe procedures for identifying introgressed genes, their paralogs, and their alleles. We also detail steps for analyzing and comparing their responses to abiotic stress in orchids.

For complete details on the use and execution of this protocol, please refer to Jiao et al.^1^

## Highlights

• Steps for the genome-wide identification of introgressed genes
• Instructions for identifying introgressed genes involved in abiotic stress responses
• Framework for evaluating the functional roles of ancient introgression

## Abstract (editor highlights)

Publisher’s note: Undertaking any experimental protocol requires adherence to local institutional guidelines for laboratory safety and ethics.

## Graphical abstract

**Figure:**
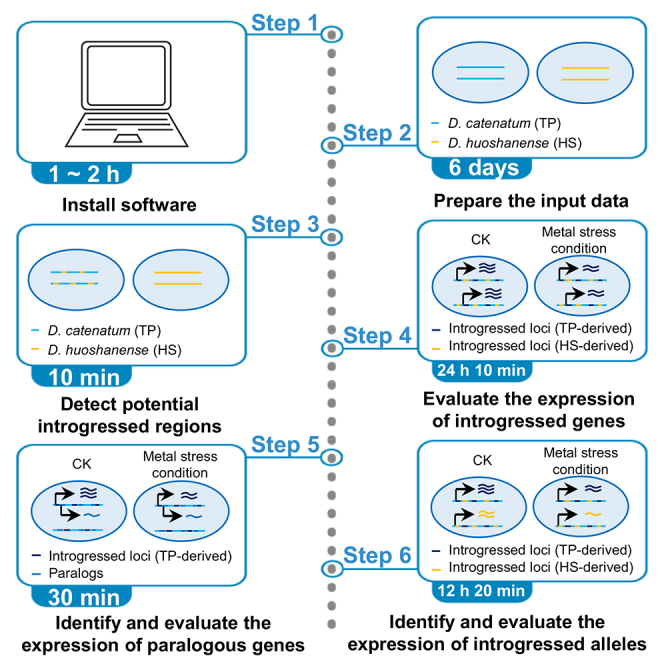

## Before you begin

Introgressed segments derived from exogenous species often encompass gene clusters that play a significant role in shaping the evolutionary trajectories of recipient species as they adapt to new habitats.^2^ However, the rapid degradation of archaic DNA and RNA in nature,^3^ along with the fact that many introgressed genes may be selected against and gradually lost over time,^4^ limits functional genomic insights into recipient species. Nevertheless, the ancient introgressed genomic segments retained in contemporary species provide a valuable opportunity to investigate the functional implications of introgression, particularly in perennial herbaceous plants.^1^

Apart from introgression, most eukaryotic genomes contain a substantial fraction of duplicated genes.^5^^,^^6^ According to previous studies, the mechanisms driving the retention of duplicated genes or paralogs may be influenced by gene dosage effects or by functional divergence through asymmetric evolution.^7^ Retention of duplicates may result from subfunctionalization, leading to a pair of genes that together perform the full range of functions of the single ancestral gene.^8^^,^^9^ Evidence of functional partitioning of ancestral functions includes, but is not limited to, biochemical functions^10^ and gene expression regulatory elements.^11^ Considering the potential functional similarity among introgressed genes, their paralogs, and alleles in recipient species, it is necessary to identify their paralogs and alleles and compare their relative expression under abiotic stress conditions. The protocol outlines the steps for detecting ancient introgressed genes between distantly related species and introduces a flexible method for identifying paralogs and alleles of these introgressed genes.

### Innovation

The protocol additionally provides methodologies for investigating the potential functional implications of introgressed genes through integration of differential expression analyses performed under abiotic stress conditions, coupled with comparative evaluations of the expression profiles of introgressed genes in relation to their paralogs and alleles. Using this protocol, we can enhance the identification of ancient introgressed loci in contemporary species and more accurately assess the potential functional roles of introgressed genes by comparing them with their paralogs and alleles in response to abiotic stress.

### Preparation 1: Hardware

1. All tests are conducted on a system running the CentOS Linux 7 (Core) operating system with Intel® Xeon® Gold 6348H CPUs operating at 2.30 GHz and supported by 1 TB of RAM.

### Preparation 2: Setting up the software

**Timing: 1–2 h**

**Figure 1:**
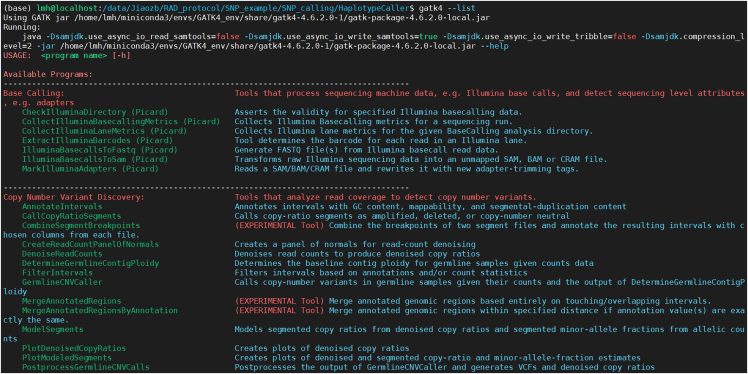
Screenshot displaying the help message of GenomeAnalysisTK The figure displays the help information for GATK4, including the version, background details, and usage instructions for the package.

In this protocol, all tests are conducted utilizing software, as detailed in the key resources table, with download links provided for access. It is recommended to store all software files in a designated folder. Additionally, software can be downloaded and installed either manually or through Conda using the default settings (Figure 1).

2. Prior to the bioinformatics analysis, install all software specified in the key resources table.

***Note:*** Installation instructions for each program are available on their official websites.

### Preparation 3: Dataset

**Timing: Variable (affected by dataset size and computational resources)**

3. Obtain raw resequencing data.

To facilitate more efficient downloading and management of raw resequencing data, install the SRA Toolkit and organize the raw data files within a designated directory. This protocol utilizes Restriction Site-Associated DNA sequencing (RAD-seq) datasets from populations of *Dendrobium catenatum* and *D. huoshanense*, as well as transcriptomic data of *D. catenatum* subjected to various cadmium stress conditions, all obtained from publicly available sources. Details regarding the download of these data can be found in the key resources table.

## Key resources table

**Table:** 

REAGENT or RESOURCE	SOURCE	IDENTIFIER
**Deposited data**		

RAD-seq data	Jiao et al.^1^	CNGBdb (CNP0008137)
*D. huoshanense* genome	Han et al.^12^	CNGBdb (CNP0000830)
Transcriptomic data of *D. catenatum*	Jiang et al.^13^	NCBI (PRJNA561268)

**Software and algorithms**		

GATK4 v4.6.2.0–1	McKenna et al.^14^	https://github.com/broadinstitute/gatk/releases
parseVCF.py	Martin et al.^15^	https://github.com/simonhmartin/genomics_general/tree/master/VCF_processing/parseVCF.py
ABBABABAwindows.py	Martin et al.^15^	https://github.com/simonhmartin/genomics_general/blob/master/ABBABABAwindows.py
ncbi-blast-2.14.0+	Altschul et al.^16^	https://ftp.ncbi.nlm.nih.gov/blast/executables/blast+/
KofamKOALA	Aramaki et al.^17^	https://www.genome.jp/tools/kofamkoala/
MEGA v5.2	Tamura et al.^18^	https://www.megasoftware.net/
FastQC v0.12.1	Andrews^19^	https://www.bioinformatics.babraham.ac.uk/projects/fastqc/
Agat v0.8.1	Dainat et al.^20^	https://github.com/NBISweden/AGAT/tree/v0.8.1
Trim_galore v0.6.10	Krueger et al.^21^	https://github.com/FelixKrueger/TrimGalore/tree/0.6.10
Hisat2 v2.2.1	Kim et al.^22^	https://github.com/DaehwanKimLab/hisat2
Samtools v1.20	Li et al.^23^	https://samtools.sourceforge.net/
HTSeq v0.11.3	Anders et al.^24^	https://github.com/htseq/htseq
DESeq2	Love et al.^25^	https://github.com/galaxyproject/tools-iuc/tree/main/tools/deseq2
ggplot2 v4.0.1	Valero-Mora^26^	https://github.com/tidyverse/ggplot2/
FastTree v2.1.11	Price et al.^27^	http://microbesonline.org/fasttree
FigTree v1.4.3	Rambaut^28^	http://tree.bio.ed.ac.uk/
TBtools v2.357	Chen et al.^29^	https://github.com/CJ-Chen/TBtools-II

**Other**		

SRA Toolkit v2.10.0	NCBI	https://github.com/ncbi/sra-tools
R v4.5.1	R Core Team	https://www.r-project.org/
Origin v2025b	OriginLab	https://www.originlab.com/demodownload.aspx

## Step-by-step method details

### Prepare input genotype data for analysis

**Timing: 6 days**

This step involves SNP calling for all individuals studied and prepares a VCF file for downstream analysis.

1. Variant calling with GATK HaplotypeCaller.

**Figure 2:**
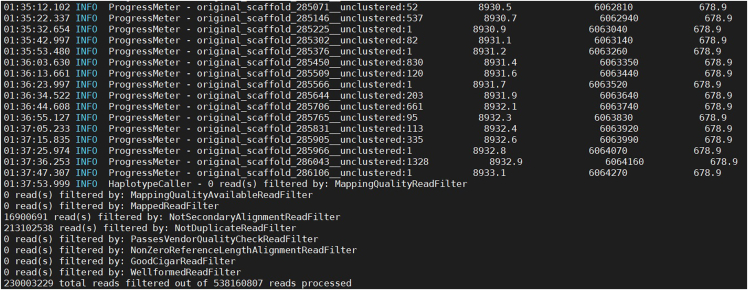
Screenshot displaying information about the SNP calling process The figure displays the process and summary information of SNP calling for all twenty tested individuals using GATK4.

>gatk4 HaplotypeCaller -R /home/[user name]/Workfiles/SNP_Calling/reference.fasta -O /home/[user name]/Workfiles/SNP_Calling/example.raw.vcf -I /home/[user name]/Workfiles/Raw_data/Sample01.realign.bam -I /home/[user name]/Workfiles/Raw_data/Sample02.realign.bam -I /home/[user name]/Workfiles/Raw_data/Sample03.realign.bam … -I /home/[user name]/Workfiles/Raw_data/SampleXX.realign.bam

***Note:*** This section identifies variants for each individual using the HaplotypeCaller module in GATK4 v4.6.2.0-1^14^ (Figure 2). To conduct a preliminary evaluation of this protocol, we obtained and analyzed genotype data files from twenty samples across three species: *D. catenatum* (ten individuals), *D. huoshanense* (five individuals), and *Flickingeria albopurpurea* (five individuals). We selected the *D. catenatum* genome as the reference genome. Troubleshooting 1.

2. Selecting SNP variations using GATK.

>gatk4 SelectVariants -select-type SNP -V /home/[user name]/Workfiles/SNP_Calling/example.raw.vcf -O /home/[user name]/Workfiles/SNP_Calling/example.raw.SNP.vcf

3. Variant filtering with GATK.

**Figure 3:**
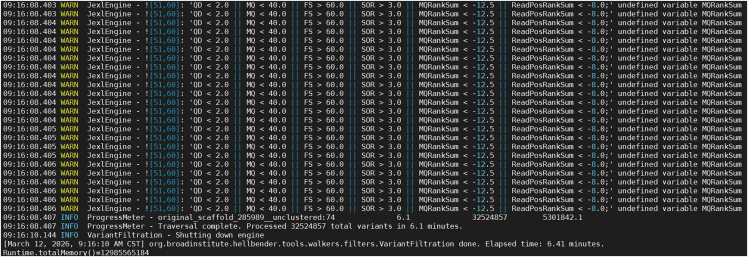
Screenshot displaying information about the SNP filtering process The figure displays the process and summary information of SNP filtering for all twenty tested individuals using GATK4.

>gatk4 VariantFiltration -R /home/[user name]/Workfiles/SNP_Calling/reference.fasta -V /home/[user name]/Workfiles/SNP_Calling/example.raw.SNP.vcf --filter-expression "QD < 2.0 || MQ < 40.0 || FS > 60.0 || SOR > 3.0 || MQRankSum < -12.5 || ReadPosRankSum < -8.0" --filter-name "Filter" -O /home/[user name]/Workfiles/SNP_Calling/example.filtered.SNP.vcf

***Note:*** This section performs SNP filtering using the VariantFiltration module in GATK4 v4.6.2.0-1^14^ (Figure 3). After filtering, convert the resulting VCF file into a ∗.geno format file using the script parseVCF.py.

4. Parsing VCF files.

>python parseVCF.py -i /home/[user name]/Workfiles/SNP_Calling/example.filtered.vcf.gz --minQual 30 --skipIndels --gtf flag=DP min=3 | gzip > /home/[user name]/Workfiles/SNP_Calling/output.example.filtered.vcf.geno.gz

### Detect potential introgressed regions

**Timing: 10 min**

This section runs the ABBA-BABA pipeline to compute the *f*_dM_ statistic and identify potential introgressed regions.

5. Compute ABBA-BABA statistics using sliding windows.

**Figure 4:**
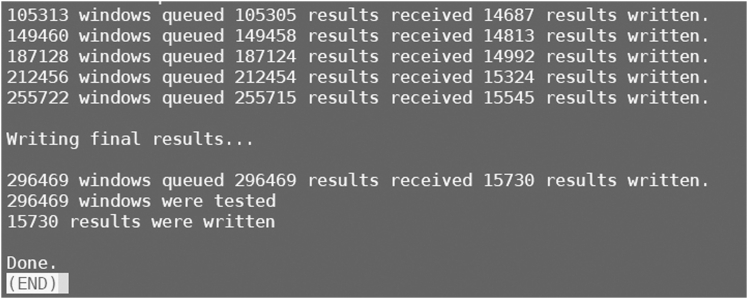
Screenshot displaying information about the analysis process of ABBABABAwindows.py The figure displays the process and summary information of introgression analysis using ABBABABAwindows.py on the genotype dataset of all tested individuals.

>python /home/[user name]/Workfiles/FdM_statistics/Script/ABBABABAwindows.py -g /home/[user name]/Workfiles/FdM_statistics/example.filtered.vcf.geno.gz -f phased -o /home/[user name]/Workfiles/FdM_statistics/output.example.filtered.vcf.geno.csv -w 10000 -m 100 -s 10000 -P1 popA P_A_1_, P_A_2_, P_A_3_, P_A_4_ -P2 popB P_B_1_, P_B_2_, P_B_3_, P_B_4_ -P3 popC P_C_1_, P_C_2_, P_C_3_, P_C_4_ -O outgroup P_O_1_, P_O_2_, P_O_3_, P_O_4_ -T 10 --minData 0.5

***Note:*** This section employs the *f*_dM_ introgression statistic to estimate shared genetic variation between the two species or groups within each genomic sliding window (Figure 4). For gene flow analysis, genotype data and sample information specifying species or groups are required for the ABBA/BABA pipeline^15^ to test for introgressed loci. Troubleshooting 2.

### Evaluate the response of introgressed genes to cadmium stress

**Timing: 24 h 10 min (step 6 to step 13)**

**Timing: 30 min (step 6)**

**Timing: 9 h (step 7)**

**Timing: 30 min (step 8)**

**Timing: 1 h (step 9)**

**Timing: 2 h (step 10)**

**Timing: 2 h (step 11)**

**Timing: 9 h (step 12)**

**Timing: 10 min (step 13)**

Transcriptome analysis is a fundamental approach for understanding the functional of genes under various conditions. This section presents a transcriptome analysis to evaluate the relative expression levels of introgressed genes under both control and stress conditions.

6. Perform a comprehensive quality assessment of the raw RNA sequencing data.

**Figure 5:**
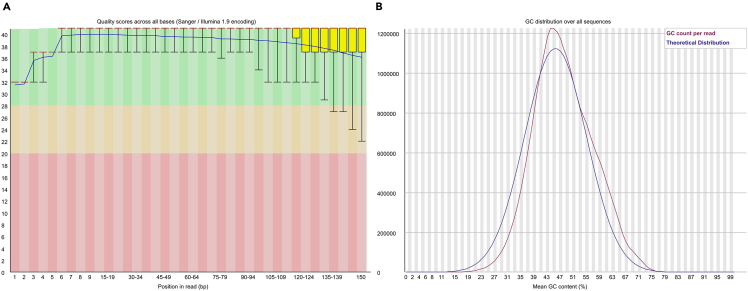
Example of quality evaluation results for sample data using FASTQ The figure presents the quality assessment of raw RNA sequencing data from a *Dendrobium catenatum* individual under control conditions (CK1). (A) Quality scores across all bases. The upper and lower whiskers represent the range of quality scores from the 10th to the 90th percentile. (B) GC content distribution across all sequences.

>fastqc -t 10 /home/[user name]/Workfiles/Raw_data/${sample}_1.fastq.gz /home/[user name]/Workfiles/Raw_data/${sample}_2.fastq.gz –o /home/[user name]/Workfiles/01.FastQC_output

***Note:*** FastQC^19^ is a widely used software tool for this purpose and can be installed via Conda. This section runs FastQC^19^ to assess the quality of sequencing read data (Figure 5). Repeat this procedure for the remaining samples.

7. Trim low-quality reads using trim_galore.

>trim_galore --paired --quality 25 --stringency 5 /home/[user name]/Workfiles/Raw_data/${sample}_1.fastq.gz /data /home/[user name]/Workfiles/Raw_data/${sample}_2.fastq.gz -o /home/[user name]/Workfiles/02.Trim_galore_output

***Note:*** This section runs the Trim_Galore^21^ tool to filter reads containing low-quality bases. Repeat this procedure for the remaining samples. Among the four output files generated by Trim_Galore, the paired ∗.fq.gz files can be used for alignment in subsequent steps.

8. Run quality control again on the trimmed files.

**Figure 6:**
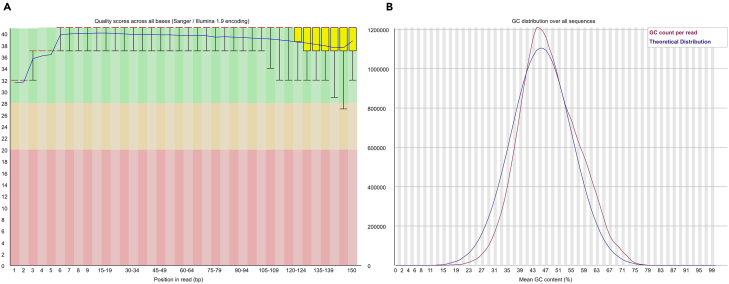
Example of quality evaluation results of post-trimming sample data using FASTQ The figure displays the quality assessment results of post-trimming RNA sequencing data from a *Dendrobium catenatum* individual under control conditions (CK1). (A) Quality scores across all bases. The upper and lower whiskers represent the range of quality scores from the 10th to the 90th percentile. (B) GC content distribution across all sequences.

>fastqc -t 10 /home/[user name]/Workfiles/02.Trim_galore_output/${sample}_1_val_1.fq.gz /home/[user name]/Workfiles/02.Trim_galore_output/${sample}_2_val_2.fq.gz –o /home/[user name]/Workfiles/03.FastQC_output

***Note:*** After filtering out low-quality bases, reassess the quality of the filtered transcriptome read data to ensure its accuracy and reliability. This section runs FastQC^19^ to evaluate the quality of the filtered sequencing reads (Figure 6). Repeat this procedure for the remaining samples.

9. Index of relevant genome.

>cd /home/[user name]/Workfiles/Reference/

>agat_convert_sp_gff2gtf.pl --gff Reference.gff -o Reference.gtf

>extract_exons.py Reference.gtf > Reference.exons.gtf

>extract_splice_sites.py Reference.gtf > Reference.splice_sites.txt

>hisat2-build --ss Reference.splice_sites.txt --exon Reference.exons.gtf Reference.fasta Reference

>cp Reference∗ /home/[user name]/Workfiles/04.Genomic_index_output/

***Note:*** This section uses the recipient species’ genome as the reference and employs HISAT2^22^ to construct the genome index. We use the genome of *D. catenatum* (tiepishihu) as the reference.

10. Align the transcriptomic sequences to the reference.

**Figure 7:**
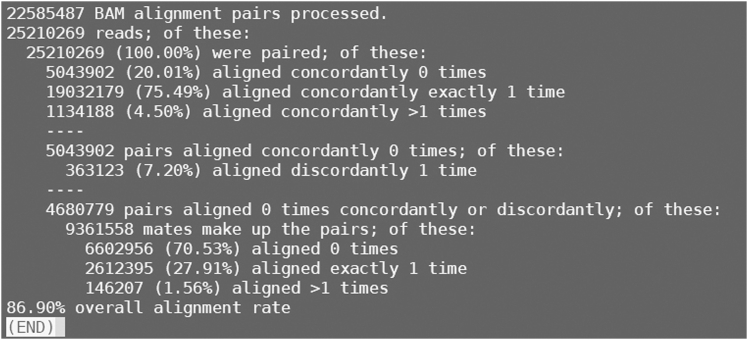
Screenshot displaying information about the analysis process of hisat2 The figure illustrates the analysis process and summarizes the transcriptomic read data from a *Dendrobium catenatum* sample, aligned to their respective genes in the reference genome using HISAT2.

>hisat2 --dta --thread 10 -x /home/[user name]/Workfiles/04.Genomic_index_output/Reference -1 /home/[user name]/Workfiles/02.Trim_galore_output/${sample}_1_val_1.fq.gz -2 /home/[user name]/Workfiles/02.Trim_galore_output/${sample}_2_val_2.fq.gz -S /home/[user name]/Workfiles/05.Hisat2_output/${sample}_aligned.sam

***Note:*** After removing low-quality bases, this step uses HISAT2^22^ to accurately align transcriptome sequencing reads to their corresponding genes in the reference genome (Figure 7). Repeat this procedure for the remaining samples. The output file from HISAT2^22^ is a tab-delimited SAM format file that can be used in subsequent steps.

11. Conversion SAM file to BAM file.
  a. Convert SAM files to BAM format using Samtools.
    >samtools view -@ 10 -bS /home/[user name]/Workfiles/05.Hisat2_output/${sample}_aligned.sam -o /home/[user name]/Workfiles/05.Hisat2_output/${sample}_aligned.bam
  b. Reordering BAM files using Samtools.
    >samtools sort -@ 10 /home/[user name]/Workfiles/05.Hisat2_output/${sample}_aligned.bam -o /home/[user name]/Workfiles/05.Hisat2_output/${sample}_sort_aligned.bam
  c. Indexing BAM files using Samtools.
    >samtools index /home/[user name]/Workfiles/05.Hisat2_output/${sample}_sort_aligned.bam
    ***Note:*** This section uses the Samtools software suite^23^ to convert the SAM file to BAM format, then sorts and indexes the resulting BAM files.
12. Quantify expressed genes and transcripts.

>htseq-count -r pos -f bam -s no -t exon /home/[user name]/Workfiles/05.Hisat2_output/${sample}_sort_aligned.bam /home/[user name]/Workfiles/04.Genomic_index_output/Reference.gtf > /home/[user name]/Workfiles/06.Htseq_output/${sample}_counts.txt

***Note:*** This section employs HTSeq^24^ to calculate read counts per gene. After repeating this process for the remaining samples, the output file ${sample}_counts.txt is a text file that requires further processing. Each text file should be imported in Excel, retaining only the “GeneID” and “Counts per sample” columns.^30^ The output files from all samples should then be consolidated by arranging their respective “Counts per sample” columns side-by-side in a single spreadsheet.^30^ The Excel file containing the “GeneID” and “Counts per sample” data is necessary for differential expression analysis using DESeq2.^25^

13. Differential expression analysis.

**Figure 8:**
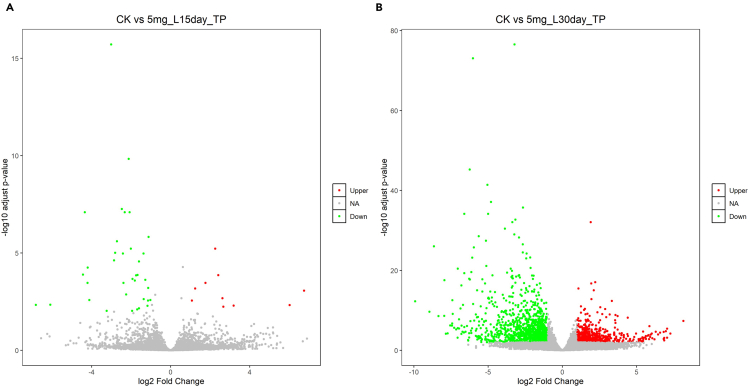
Volcano plots illustrating the log2-fold change in gene expression under control (CK) and cadmium stress conditions, using the *D. catenatum* genome as a reference (A) Graphs depicting the alterations in gene expression between the control group (CK) and the 5mg/L cadmium treatment after 15 days (T15D). (B) Graphs depicting the alterations in gene expression between the control group (CK) and the 5mg/L cadmium treatment after 30 days (T30D).

>setwd(“/Users/[user name]/DESeq2_workfile")

>dat <- read.csv("CK_T15D_TP_counts_s.csv")

>col_Data <- data.frame(condition = factor(rep(c('CK','T15D'),each=3),level=c('CK','T15D')))

>library(DESeq2) # Load DESeq2

>Differ <- DESeqDataSetFromMatrix(countData = dat, colData = col_Data, design= ∼condition)

>Differ01 <- DESeq(Differ, fitType = 'mean', minReplicatesForReplace = 7, parallel = FALSE)

>result <- results(Differ01, contrast = c('condition', 'CK', 'T15D'))

>result01 <- data.frame(result, stringsAsFactors = FALSE, check.names = FALSE)

>write.table(result01, 'CK-5mg_L15day_TP.DESeq2.result', col.names = NA, quote = FALSE, sep = '∖t')

>result01 <- result01[order(result01$padj, result01$log2FoldChange, decreasing = c(FALSE, TRUE)), ]

>result01[which(result01$log2FoldChange >= 1 & result01$padj < 0.01),'sig'] <- 'Upper'

>result01[which(result01$log2FoldChange <= -1 & result01$padj < 0.01),'sig'] <- 'Down'

>result01[which(abs(result01$log2FoldChange) <= 1 | result01$padj >= 0.01),'sig'] <- 'NA'

>res1_selected <- subset(result01, sig %in% c('Upper', 'Down'))

>write.table(res1_selected, file = 'CK-5mg_L15day_TP.selected.result', quote = FALSE, sep = '∖t')

>result01_U <- subset(result01, sig == 'Upper')

>result01_D <- subset(result01, sig == 'Down')

>write.table(result01_U, file = 'CK-5mg_L15day_TP.selected.up.result', quote = FALSE, sep = '∖t')

>write.table(result01_D, file = 'CK-5mg_L15day_TP.selected.down.result', quote = FALSE, sep = '∖t')

>library(ggplot2)

>plot <- ggplot(data = result01, aes(x = log2FoldChange, y = -log10(padj), color = sig)) + geom_point(size = 1)+scale_color_manual(values = c('red', 'gray', 'green'), limits = c('Upper', 'NA', 'Down')) +labs(x = 'log2 Fold Change', y = '-log10 adjust p-value', title = 'CK vs 5mg_L15day_TP', color = ") + theme(plot.title = element_text(hjust = 0.5, size = 14), panel.grid = element_blank(), panel.background = element_rect(color = 'black', fill ='transparent'),legend.key = element_rect(fill = 'transparent'))

>ggsave(plot, filename = "CK-5mg_L15day_TP.png")

***Note:*** DESeq2^25^ is an R package to analyze differentially expressed genes and generate graphical representations of the data. This section employs DESeq^25^ to identify differentially expressed genes, using a significance threshold of an absolute log2 fold change greater than 1 and an adjusted *p*-value less than 0.01. In this section, a control group (CK) and two treatment groups—5 mg/L cadmium for 15 days (T15D) and 5 mg/L cadmium for 30 days (T30D)—are compared (Figure 8).

### Identify paralogs of introgressed genes associated with metal stress response

**Timing: 30 min**

This section uses BLASTP and KofamKoALA to identify paralogs of introgressed genes involved in the response to stress conditions.

14. Retrieve the protein sequences of the introgressed genes in FASTA format.
  This section focuses on introgressed genes that are implicated in the response to cadmium induced stress. The approach involves comparing these introgressed genes with a dataset of differentially expressed genes, identifying those that exhibit differential expression in response to cadmium induced stress, and subsequently extracting their corresponding protein sequences to serve as query sequences for downstream analyses.
  a. Indexed the complete protein sequence file of the reference genome.
    ***Note:*** This section uses the recipient species’ genome as the reference and employs makeblastdb to construct the localized database. The protein sequences were extracted from the *D. catenatum* genome to construct the localized database.
    >makeblastdb -in /home/[user name]/Workfiles/Protain_database_recipient/Reference_protein_sequence.fas -dbtype prot -parse_seqids
  b. BLAST search of a protein sequence against the local database.
    >blastp -query /home/[user name]/Workfiles/Protain_database_recipient/Candidate_introgressed_genes_pep.fasta -db /home/[user name]/Workfiles/Protain_database_recipient/Reference_protein_sequence.fas -out /home/[user name]/Workfiles/Protain_database_recipient/Candidate_introgressed_genes_blastp_Reference_genome_pep.out -outfmt 6
    ***Note:*** This section uses BLASTP^16^ to extract protein sequences of introgressed genes that exhibit differential expression under stress. We extracted the protein sequences of introgressed genes exhibiting differential expression under stress and used them as queries to search for paralogs in the reference local database, applying an E-value threshold of less than 1.0E-05.
  c. Functional annotation of proteins.
    This section uses KofamKoALA^17^ to annotate the remaining homologous proteins and selects those with consistent annotation information.
15. Detect the relationships among homologous proteins.
  a. After obtaining the homologous gene for each candidate introgressed gene, align them using MEGA v5.2.2,^18^ and generate an alignment file of their amino acid sequences.
  b. Using the aligned protein sequence file, perform a maximum likelihood analysis with FastTree v2.1.1^27^ using the default settings, and generate a tree file for constructing the phylogenetic figure.
  c. Using FigTree v1.4.3^28^ to visualize the phylogenetic tree, paralogs grouped with the query introgressed genes were selected.

### Identify the introgressed alleles and analyze their expression in response to metal-induced stress

**Timing: 12 h 20 min (step 16 to step 20)**

**Timing: 10 min (step 16)**

**Timing: 2 h (step 17)**

**Timing: 1 h (step 18)**

**Timing: 9 h (step 19)**

**Timing: 10 min (step 20)**

This section uses BLASTP and KofamKoALA to identify orthologs of introgressed genes involved in the response to stress conditions, and selects the most appropriate ortholog as the introgressed allele derived from the donor species.

16. Retrieve the protein sequences of the introgressed alleles in FASTA format.
  a. Indexed the complete protein sequence file of the donor species’ genome.
    >makeblastdb -in /home/[user name]/Workfiles_D/Protain_database_Donor/donor_genome_pep.fas -dbtype prot -parse_seqids
    ***Note:*** This section uses the donor species’ genome as the reference and employs makeblastdb to construct the localized database. The protein sequences were extracted from the *D. huoshanense* genome to construct the localized database.
  b. BLAST search of a protein sequence against the local database.
    >blastp -query /home/[user name]/Workfiles_D/Protain_database_Donor/Candidate_introgressed_genes_pep.fasta -db /home/[user name]/Workfiles_D/Protain_database_Donor/Reference_genome_pep.fasta -out /home/[user name]/Workfiles_D/Protain_database_Donor/Candidate_introgressed_genes_blastp_Reference_genome_pep.out -outfmt 6
    ***Note:*** To identify orthologous genes, use the protein sequences of the introgressed genes as query inputs to search the genome database of the donor species. Employ BLASTP^16^ to identify candidate orthologs, applying an E-value threshold of less than 1.0E-40.
  c. Functional annotation of proteins.
    i. After filtering, use KofamKoALA^17^ to annotate the remaining homologous proteins and verify the functional information of the candidate orthologs.
    ii. According to the KofamKoALA results, identify the orthologous gene with the lowest E-value and consistent functional annotation as the most appropriate ortholog, and select it as the introgressed allele derived from the donor species.
17. Align the transcriptomic sequences to the donor species’ reference genome.
  a. Index the reference genome.
    >agat_convert_sp_gff2gtf.pl --gff Reference_donor.gff -o Reference_donor.gtf
    >extract_exons.py Reference_donor.gtf > Reference_donor.exons.gtf
    >extract_splice_sites.py Reference_donor.gtf > Reference_donor.splice_sites.txt
    >hisat2-build --ss Reference_donor.splice_sites.txt --exon Reference_donor.exons.gtf Reference_donor.fasta Reference_donor
  b. Align the transcriptomic sequencing reads to the reference genome.
    >hisat2 --dta --thread 10 -x /home/[user name]/Workfiles_D/Reference_Dhuo/Reference_donor -1 /home/[user name]/Workfiles/02.Trim_galore_output/${sample}_1_val_1.fq.gz -2 /home/[user name]/Workfiles/02. Trim_galore_output/${sample}_2_val_2.fq.gz -S /home/[user name]/Workfiles_D/05.Hisat2_output/${sample}_aligned.sam
    ***Note:*** This section selects the donor species’ genome as the reference and uses HISAT2^22^ to generate indexed files. Repeat this process for the remaining samples.
18. Conversion SAM file to BAM file.
  a. Convert SAM files to BAM format using Samtools.
    >samtools view -@ 10 -bS /home/[user name]/Workfiles_D/05.Hisat2_output/${sample}.sam -o /home/[user name]/Workfiles_D/05.Hisat2_output/${sample}.bam
  b. Reordering BAM files using Samtools.
    >samtools sort -@ 10 /home/[user name]/Workfiles_D/05.Hisat2_output/${sample}.bam -o /home/[user name]/Workfiles_D/05.Hisat2_output/${sample}.sort.bam
  c. Indexing BAM files using Samtools.
    >samtools index /home/[user name]/Workfiles_D/05.Hisat2_output/${sample}.sort.bam
    ***Note:*** After aligning transcriptomic sequences to the donor species’ reference genome, the output file generated by HISAT2^22^ is in SAM format. This step uses the Samtools software suite^23^ to convert the SAM format file to BAM format, then sorts and indexes the BAM file for subsequent analysis. Repeat this process for the remaining samples.
19. Quantify expressed genes and transcripts.

>htseq-count -r pos -f bam -s no -t exon /home/[user name]/Workfiles_D/05.Hisat2_output/${sample}_sort_aligned.bam /home/[user name]/Workfiles_D/Reference_Dhuo/huoshanshihu.gtf > /home/[user name]/Workfiles_D/06.Htseq_output/${sample}_counts.txt

***Note:*** This section uses htseq-count to quantify expressed genes and transcripts. After repeating this process for the remaining samples, the output file ${sample}_counts.txt is a text file that requires further processing. Each text file should be imported into Excel, retaining only the “GeneID” and “Counts per sample” columns.^30^ The output files from all samples should then be consolidated by arranging their respective “Counts per sample” columns side-by-side in a single spreadsheet.^30^ The Excel file containing the “GeneID” and “Counts per sample” data is necessary for differential expression analysis using DESeq2.^25^

20. Differential expression analysis.

**Figure 9:**
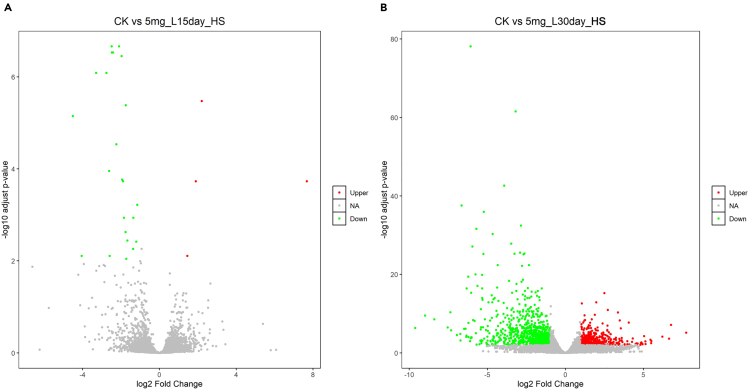
Volcano plots illustrating the log2-fold change in gene expression under control (CK) and cadmium stress conditions, using the *D. huoshanense* genome as a reference (A) Graphs depicting the alterations in gene expression between the control group (CK) and the 5mg/L cadmium treatment after 15 days (T15D). (B) Graphs depicting the alterations in gene expression between the control group (CK) and the 5mg/L cadmium treatment after 30 days (T30D).

>setwd(“/Users/[user name]/DESeq2_workfile")

>dat <- read.csv("CK_T15D_HS_counts_s.csv")

>col_Data <- data.frame(condition = factor(rep(c('CK','T15D'),each=3),level=c('CK','T15D')))

>library(DESeq2)

>Differ <- DESeqDataSetFromMatrix(countData = dat, colData = col_Data, design= ∼condition)

>Differ01 <- DESeq(Differ, fitType = 'mean', minReplicatesForReplace = 7, parallel = FALSE)

>result <- results(Differ01, contrast = c('condition', 'CK', 'T15D'))

>result01 <- data.frame(result, stringsAsFactors = FALSE, check.names = FALSE)

>write.table(result01, 'CK-5mg_L15day_HS.DESeq2.txt', quote = FALSE, sep = '∖t')

>result01 <- result01[order(result01$padj, result01$log2FoldChange, decreasing = c(FALSE, TRUE)), ]

>result01[which(result01$log2FoldChange >= 1 & result01$padj < 0.01),'sig'] <- 'Upper'

>result01[which(result01$log2FoldChange <= -1 & result01$padj < 0.01),'sig'] <- 'Down'

>result01[which(abs(result01$log2FoldChange) <= 1 | result01$padj >= 0.01),'sig'] <- 'NA'

>res1_selected <- subset(result01, sig %in% c('Upper', 'Down'))

>write.table(res1_selected, file = 'CK-5mg_L15day_HS.selected.result', quote = FALSE, sep = '∖t')

>result01_U <- subset(result01, sig == 'Upper')

>result01_D <- subset(result01, sig == 'Down')

>write.table(result01_U, file = 'CK-5mg_L15day_HS.selected.up.result', quote = FALSE, sep = '∖t')

>write.table(result01_D, file = 'CK-5mg_L15day_HS.selected.down.result', quote = FALSE, sep = '∖t')

>library(ggplot2)

>plot <- ggplot(data = result01, aes(x = log2FoldChange, y = -log10(padj), color = sig)) + geom_point(size = 1)+scale_color_manual(values = c('red', 'gray', 'green'), limits = c('Upper', 'NA', 'Down')) +labs(x = 'log2 Fold Change', y = '-log10 adjust p-value', title = 'CK vs 5mg_L15day_HS', color = '') + theme(plot.title = element_text(hjust = 0.5, size = 14), panel.grid = element_blank(), panel.background = element_rect(color = 'black', fill ='transparent'),legend.key = element_rect(fill = 'transparent'))

>ggsave(plot, filename = "CK-5mg_L15day_HS.png")

***Note:*** This step uses the DESeq2^25^ and ggplot2 package to identify differentially expressed genes and generate a figure illustrating the results (Figure 9). We used DESeq^25^ to identify differentially expressed genes, applying a significance threshold of an absolute log2 fold change greater than 1 and an adjusted *p*-value less than 0.01.

21. Compare the responses of introgressed genes with those of their paralogs and alleles under cadmium stress.

This step compiles the expression data of introgressed genes involving in the stress response, along with their paralogs and alleles, into an Excel file containing “GeneID” and “Counts per sample”. Then, this step uses TBtools v2.357^29^ and Origin v2025b (OriginLab Corporation, Northampton, MA, USA) to compare the responses of introgressed genes with those of their paralogs and alleles under stress conditions, and to generate heatmaps and boxplots.

## Expected outcomes

### Geno-format file preparation

**Figure 10:**
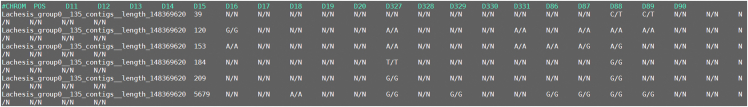
The formation and composition of the geno file The figure displays the loci information from the.geno file for all tested individuals, generated by parseVCF.py. The script’s output file contains 22 columns, including chromosome (CHROM), SNP position (POS), and unphased genotypes for each individual. Missing data are represented by ‘N’, while unphased genotypes are indicated by ‘/’.

The script’s output file contains 22 columns, including the chromosome (CHROM), SNP position (POS), and unphased genotypes for each individual (Figure 10).^15^ Missing data are represented by ‘N’, while unphased genotypes are indicated by ‘/’.^15^

### *f*_dM_ introgression statistic file

The script’s output file contains 11 columns, including the scaffold on which the window is located (scaffold), the window start position (start), the window end position (end), the window middle position (mid), the number of genotype sites within each window (sites), the number of genotype sites used to compute statistics within each window (sitesUsed), the number of ABBA sites within each window (ABBA), the pseudo count of BABA sites (BABA), the *D* statistic (*D*), the *f*_d_ admixture estimation (*f*_d_), and Malinsky’s modified statistic (*f*_dM_).^15^

### Differential expression analysis

The DESeq2 output file comprises eight columns, which include the mean of normalized counts across all samples (baseMean), the log2 fold change (log2FoldChange) representing the comparison groups under treated and untreated conditions, the standard error associated with the log2 fold change (lfcSE), the Wald test statistic (stat), the *p*-value derived from the Wald test (*p*-value), the Benjamini-Hochberg adjusted *p*-value for controlling the false discovery rate (padj), and an indicator denoting statistical significance (sig).^25^

### Identify paralogs of introgressed genes

The BLASTP output file consists of twelve columns, namely query id, subject id, percentage identity, alignment length, number of mismatches, number of gap openings, query start position (q. start), query end position (q. end), subject start position (s. start), subject end position (s. end), e-value, and bit score.^16^ A set of candidate gene members was compared against the protein sequence of the introgressed gene, and only those genes exhibiting an e-value below 1.0e−05 were selected for further analysis.

Moreover, the KofamKoALA output file contains six columns: gene name, KO, threshold, score, E-value, and KO definition.^17^ Only genes exhibiting the same KO as the introgressed gene were retained.

The output files generated from the phylogenetic analysis, conducted using FastTree version 2.1.11^27^ and subsequently visualized with Figtree version 1.4.3,^28^ are provided. These files facilitate the visualization of the phylogenetic relationships between the retained paralogs and the query introgressed gene.

### Identify alleles introgressed from the donor species

The BLASTP output file consists of twelve columns, namely query id, subject id, percentage identify, alignment length, number of mismatches, number of gap openings, query start position (q. start), query end position (q. end), subject start position (s. start), subject end position (s. end), e-value, and bit score.^16^ A set of candidate gene members was compared against the introgressed gene, and only those genes exhibiting an e-value below 1.0e−40 were selected for further analysis.

The output file generated by KofamKoALA comprises six columns: gene name, KO identifier, threshold, score, E-value, and KO definition.^17^ Only those genes that share the identical KO designation as the introgressed gene were retained. The ortholog deemed most appropriate was identified based on the lowest e-value obtained from the BLASTP analysis and congruence with the KofamKOALA results for the introgressed genes. This ortholog was subsequently designated as the introgressed allele originating from the donor species.

## Limitations

### The limited application of *D*-statistic analysis

The *D* statistic is derived from a model comprising four species or populations, which incorporates an outgroup species.^15^ This method requires four species or populations: P1, P2, P3 and O. Importantly, it assumes that individuals sampled from the outgroup population have experienced negligible admixture from the archaic source.^15^ Moreover, the model designs limitations—typically confined to four taxa—make it unsuitable for identifying introgression across broader evolutionary scales, especially in large, complex phylogenies.

### Limitations in identifying alleles from the donor species

The methods used to identify introgressed alleles derived from *D. huoshanense*—including BLASTP and KofamKoALA—as well as the evaluation of the response of these introgressed alleles to cadmium stress, do not detect all introgressed material. This is because some introgressed genes exhibit a distant relationship with their respective alleles. Generally, after prolonged selection within hybrid populations, only a small fraction of the retained introgressed genetic variation is found to be adaptive. Furthermore, after multiple rounds of backcrossing, introgressed variation may continue to be subject to purifying selection, leading to their gradual reduction over time.^4^ The remaining introgressed genes will show varying degrees of divergence from their respective alleles. If an introgressed gene exhibits significant divergence from its allele, its sequences similarity will likely be low, making it undetectable by our method.

## Troubleshooting

### Problem 1

**Figure 11:**
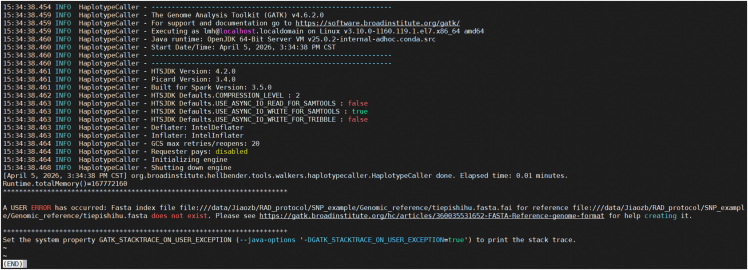
Error information from GATK The figure shows that the commands are falling because the FASTA index file for the reference genome does not exist during the SNP calling process for all tested individuals using GATK4.

Relate to variant calling with GATK HaplotypeCaller: GATK^14^ commands are failing because the FASTA index file for the reference genome does not exist (Figure 11).

### Potential solution

Index the reference genome using Samtools, BWA, and Picard software. The command lines are as follows.

• samtools faidx /home/[user name]/Workfiles/Reference_Index/Reference.fasta # Use the command “samtools faidx” to generate the FASTA index (fai) file for the reference genome.
• bwa index /home/[user name]/Workfiles/Reference_Index/Reference.fasta #Use the “bwa index” command to generate the index file for the reference genome.
• picard CreateSequenceDictionary R=/home/[user name]/Workfiles/Reference_Index/Reference.fasta O=/home/[user name]/Workfiles/Reference_Index/Reference.dict # Use the “picard CreateSequenceDictionary” tool to generate the dictionary file for the reference genome.

### Problem 2

**Figure 12:**
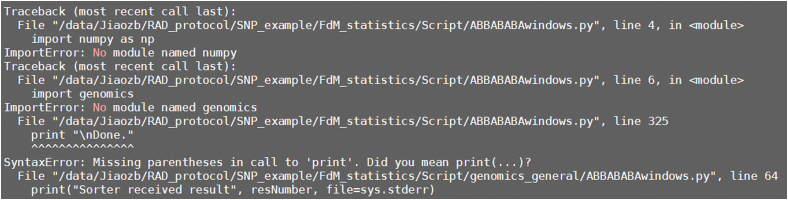
Error information from python The figure displays an error message from Python indicating that the module named ‘numpy’ is missing.

• Relate to compute ABBA-BABA statistics using sliding windows: Python commands are failing due to an ImportError: No module named ‘numpy’ (Figure 12).

### Potential solution

Install Numpy using Conda. The installation command is as follows.

• conda create -n python2.7_env # Create an environment.
• conda activate python2.7_env # Activate the environment.
• pip install numpy # Install the software into the environment.

## Resource availability

### Lead contact

Further information and requests for resources and reagents should be directed to and will be fulfilled by the lead contact, Zhong-Jian Liu (zjliu@fafu.edu.cn).

### Technical contact

Technical questions on executing this protocol should be directed to and will be answered by the technical contact, Zhong-Jian Liu (zjliu@fafu.edu.cn).

### Materials availability

This study did not generate new unique reagents.

### Data and code availability

The accession numbers for the datasets used in this paper are CNGBdb: CNP0008137 and CNP0000830, as well as NCBI: PRJNA561268. The datasets used in this paper have been listed in the key resources table. The code from this study is fully listed in this article.

## Acknowledgments

This work was supported by the Funds for Forestry Peak Discipline Construction Project of 10.13039/501100008766Fujian Agriculture and Forestry University (grant no. 72202200205).

## Author contributions

Z.-J.L., S.L., and Z.J. developed the original analysis pipeline. Z.J., Z.R., and L.-J.C. performed the analyses of the population genotypic data. Z.J. and C.H. conducted the introgression analysis. Z.J., X.M., and G.-Q.Z. carried out the transcriptome profiling and differential expression analyses. Z.J. and Z.-J.L. wrote the manuscript. Y.-B.L., S.L., D.-H.P., G.W., and Z.-J.L. provided valuable feedback on the manuscript.

## Declaration of interests

The authors declare no competing interests.
